# Disseminated Kaposi Sarcoma Associated With Cytomegalovirus Proctitis in People Living With Human Immunodeficiency Virus (PLHIV): A Major Diagnostic Dilemma

**DOI:** 10.7759/cureus.42039

**Published:** 2023-07-17

**Authors:** Rodrigo Portella, Cesar Cilento Ponce, Rosely Antunes Patzina, Jose C Ardengh, Richard Calanca

**Affiliations:** 1 Infectious Diseases, Instituto de Infectologia Emílio Ribas, São Paulo, BRA; 2 Pathology, Instituto de infectologia Emílio Ribas, São Paulo, BRA; 3 Pathology, Hospital das Clinicas da Faculdade de Medicina da Universidade de São Paulo, São Paulo, BRA; 4 Pathology, Instituto de Infectologia Emílio Ribas, São Paulo, BRA; 5 Gastrointestinal Endoscopy, Hospital das Clínicas de Ribeirão Preto, Ribeirão Preto, BRA; 6 Diagnostic Imaging, Universidade Federal de São Paulo, São Paulo, BRA; 7 Digestive Endoscopy, Instituto de Infectologia Emílio Ribas, São Paulo, BRA; 8 Digestive Endoscopy, Instituto de Infectologia Emilio Ribas, São Paulo, BRA

**Keywords:** treatment, immuno-histochemical, clinical pathology, image enhanced endoscopy, gastrointestinal manifestations, cytomegalo virus (cmv), disseminated kaposi sarcoma, hiv

## Abstract

Kaposi sarcoma (KS) is a vascular tumor of low malignancy. Lesions may vary in shape, color, and size. Angiogenesis, spindle-shaped cells, and inflammatory infiltration are the main histologic features of the condition. Human herpesvirus-8 (HHV-8) infection and immune dysfunction play a key role in the development of KS. We report a case of a 40-year-old man with disseminated KS (DKS) who underwent an endoscopic examination. Colonoscopy revealed an ulcer in the anal canal. Biopsy and immunohistochemistry (IHC) confirmed the diagnosis of cytomegalovirus (CMV) proctitis, a rare and underreported pathology.

## Introduction

Kaposi sarcoma (KS) is a multicentric vascular tumor of endothelial origin and a low-grade malignancy. Lesions may present as macules, nodules, or plaques. KS usually involves the skin and mucosa, but other sites such as lymph nodes and visceral organs may also be involved [1,2]. There are three main histologic features in KS: neoangiogenesis, spindle-shaped cells, and chronic inflammatory infiltrates [3]. The relationship between human herpesvirus-8 (HHV-8) infection and the development of KS has been well established [4]. However, the presence of HHV-8 alone is not sufficient for the development of KS, and other cofactors are required for the manifestation of this pathology [5].

Currently, four main forms of KS are known: the classic form, which is more common in certain populations of the Middle East, Eastern Europe, and the Mediterranean; the endemic form, which is more common in sub-Saharan Africa; the iatrogenic form, which results from immunosuppression induced by drugs or transplantation; and the epidemic or AIDS-related form, which is seen in HIV infection [6]. The epidemic form is the most common HIV-associated neoplasm [3], although its prevalence has decreased since the introduction of highly active antiretroviral therapy (HAART) [7].

In this report, we present a case of a patient living with HIV who was diagnosed with disseminated Kaposi sarcoma (DKS) associated with proctitis due to cytomegalovirus (CMV), also known as human herpesvirus-5 (HHV-5), an important opportunistic pathogen in HIV infection. CMV is a member of the beta-herpesvirus family and is very common worldwide, especially among adults living in poor socioeconomic conditions [8]. After retinitis, gastrointestinal manifestations are the most common CMV presentation in people living with HIV (PLHIV) [9]. DKS, characterized by multiple lesions and multisystem involvement, does not occur in the majority of cases and is more common in patients with severe immunodepression. DKS associated with CMV proctitis is even rarer, which makes this report interesting and important for daily clinical practice due to the paucity of similar reports in the literature.

## Case presentation

A 40-year-old man who has sex with men (MSM) presented with two years of diarrhea and three months of progressive dyspnea. He had been diagnosed with HIV nine years prior and had been treated with efavirenz/lamivudine/tenofovir (EFV/3TC/TDF) only for the first two months after diagnosis and had since discontinued treatment. No other comorbidities, alcohol use, tobacco use, drug use, or current medications were reported. When asked about past medical complications, the patient reported an episode of coronavirus infection without the need for hospitalization, an episode of herpes zoster treated with acyclovir, and an episode of community-acquired pneumonia in the past two years.

**Figure 1:**
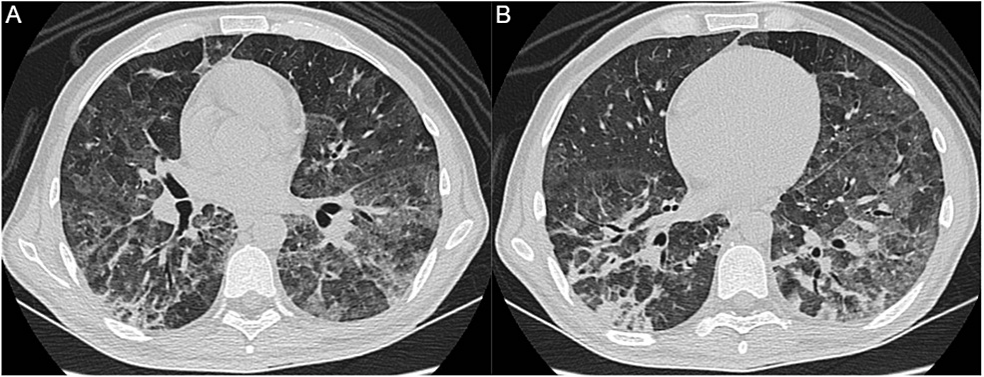
CT of the chest showing (A) ground-glass opacities in both lungs and (B) consolidation in both lower lobes CT: computed tomography

Physical examination revealed signs of acute respiratory failure with cyanosis, tachypnea, tachycardia, and a peripheral oxygen saturation of 71%. Clinical improvement was observed after the initiation of noninvasive ventilation. Laboratory results showed no anemia, leukocytosis, or platelet abnormalities. There was no renal or hepatic dysfunction. No electrolyte abnormalities were found. The lactate dehydrogenase level was elevated (578 U/L). CT of the chest showed ground-glass opacities in both lungs and consolidation in both lower lobes (Figure 1). Rapid antigen tests for influenza and COVID-19 performed on admission were both negative. Given the hypothesis of community-acquired pneumonia and Pneumocystis pneumonia (PCP), the patient was started on ceftriaxone, trimethoprim-sulfamethoxazole, and prednisone. Clinical improvement was observed in the following days. His CD4 count and viral load in plasma on admission were 7 cells per microliter and 108,098 copies per milliliter, respectively.

During hospitalization, sputum and bronchoalveolar lavage specimens were tested for tuberculosis. Xpert MTB/RIF and bacilloscopy were negative. Cultures of bronchoalveolar lavage specimens were all negative, including culture for mycobacteria and culture for fungus. Stool examination for Cryptosporidium spp., Cystoisospora belli, helminths, and protozoa was negative. Fundoscopy was performed and showed no abnormalities. Purplish macules were observed on the face, palate, buttocks, genitals, and lower extremities, suggestive of KS. A skin biopsy was performed and showed atypical vascular proliferation. Immunohistochemistry (IHC) was positive for HHV-8.

**Figure 2:**
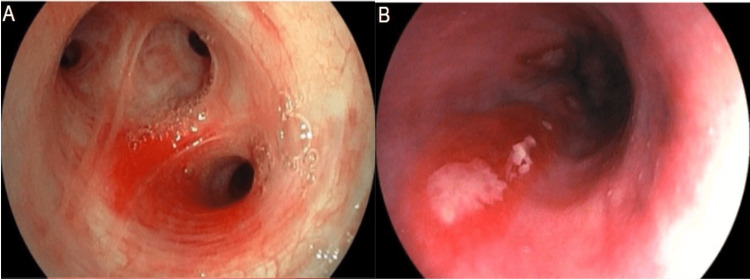
(A) Reddish lesion on right bronchus. (B) Reddish and fibrinous lesion on the esophagus

**Figure 3:**
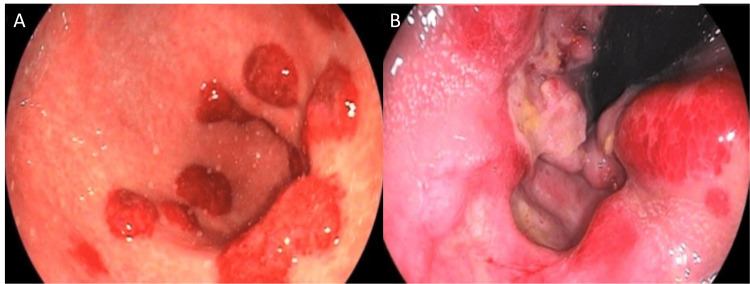
(A) Reddish and elevated lesions on the stomach. (B) Reddish lesions and extensive ulcer on the anal canal

Staging was performed with bronchoscopy, upper endoscopy, and colonoscopy, which revealed lesions suggestive of KS in the respiratory tract (Figure 2a) and gastrointestinal tract from the mouth to the anal canal (Figures 2b, 3).

**Figure 4:**
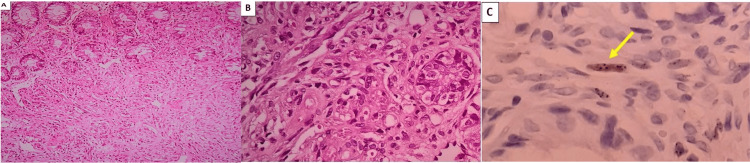
(A) Spindle cell neoplasm delimiting vascular lacunae and characterizing KS in the rectal mucosa (H&E, 40x). (B) KS area in detail (H&E, 400x). (C) HHV-8 infection in neoplastic cells (IHC LANA-1, 400x) KS: Kaposi sarcoma; HHV-8: human herpesvirus-8

**Figure 5:**
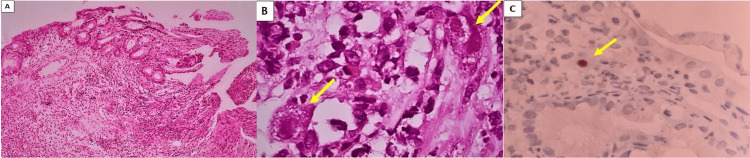
(A) CMV proctitis (H&E, 40x). (B) CMV cytopathic effect in endothelial cells: cytomegaly associated with basophilic intranuclear inclusions surrounded by a clear halo and granular eosinophilic cytoplasmic inclusions (H&E, 400x). (C) Immunohistochemistry showing endothelial cells with CMV antigen in the nuclei (IHC CMV, 400x) CMV: cytomegalovirus

Biopsy of the rectal lesion stained with hematoxylin and eosin (H&E) showed atypical vascular proliferation and immunohistochemistry was positive for HHV-8 (Figure 4). An ulcer was also found in the anal canal, which was shown by biopsy and IHC to be secondary to CMV infection (Figure 5).

Hospitalization lasted about one month. ART was restarted (tenofovir/lamivudine/dolutegravir); CMV proctitis was treated with ganciclovir, which was later switched to foscarnet due to ganciclovir-induced neutropenia. Chemotherapy for DKS was started during hospitalization. Colonoscopy was repeated after seven cycles of chemotherapy with liposomal doxorubicin and showed no lesions suggestive of KS and scarring of the CMV-induced ulcer.

## Discussion

The estimated number of PLHIV worldwide was 38.4 million in 2021, with 1.5 million new cases [9]. Although the HIV epidemic scenario has radically changed since the introduction of HAART, several challenges still remain. Diagnosis of gastrointestinal disease in PLHIV may require invasive testing [10]. In our report, a biopsy guided by endoscopic examination was fundamental in establishing the diagnosis of CMV proctitis in a patient with DKS.

KS is one of the most common neoplasms in PLHIV [11]. Although the diagnosis is often based on the characteristics of the lesions, a biopsy should be performed whenever possible to avoid misdiagnosis [12]. The need for staging must be assessed in all cases and includes mucosal inspection and endoscopic examination. The suspected involvement of the hollow viscera requires endoscopic evaluation [13]. Early cases can be treated with HAART or minimally invasive therapy, while advanced cases require systemic chemotherapy [14].

CMV proctitis appears to be a rare diagnosis. A literature review by Studemeister found only 16 cases between 1964 and 2011 [14]. CMV proctitis after anal intercourse was found in seven patients. The mean age was 29 years and all cases resolved spontaneously without complications. The remaining cases involved older patients with multiple comorbidities and worse outcomes.

The Sexually Transmitted Infections Treatment Guidelines [15] list the following pathogens as possible causes of sexually transmitted proctitis: C. trachomatis, N. gonorrhoeae, H. simplex, T. pallidum, and M. genitalium. CMV is not mentioned as a cause of sexually transmitted proctitis, probably due to the paucity of cases reported in the literature.

## Conclusions

Based on our findings, PLHIV with DKS may benefit from biopsies guided by endoscopic examination. This strategy enables the correct staging of KS and identification of concomitant pathologies, which are not uncommon in severely immunosuppressed PLHIV. The true prevalence of CMV proctitis is not known, which warrants further research into this rare and possibly underdiagnosed disease.

## References

[1] Resende C, Azevedo T, Henriques A (2015). Kaposi´s sarcoma - a clinicopathological review. Revista da SPDV.

[2] Radu O, Pantanowitz L (2013). Kaposi sarcoma. Arch Pathol Lab Med.

[3] Pantanowitz L, Dezube BJ (2008). Kaposi sarcoma in unusual locations. BMC Cancer.

[4] Ma JY, Liu JW (2022). Disseminated Kaposi sarcoma. Clin Cosmet Investig Dermatol.

[5] Mesri EA, Cesarman E, Boshoff C (2010). Kaposi's sarcoma and its associated herpesvirus. Nat Rev Cancer.

[6] Cavallin LE, Goldschmidt-Clermont P, Mesri EA (2014). Molecular and cellular mechanisms of KSHV oncogenesis of Kaposi's sarcoma associated with HIV/AIDS. PLoS Pathog.

[7] Cesarman E, Damania B, Krown SE, Martin J, Bower M, Whitby D (2019). Kaposi sarcoma. Nat Rev Dis Primers.

[8] Eltom MA, Jemal A, Mbulaiteye SM, Devesa SS, Biggar RJ (2002). Trends in Kaposi's sarcoma and non-Hodgkin's lymphoma incidence in the United States from 1973 through 1998. J Natl Cancer Inst.

[9] (2023). The Joint United Nations Programme on HIV/AIDS - 2022. https://www.unaids.org/en/2022.

[10] Olanipekun T, Kagbo-Kue S, Egwakhe A, Mayette M, Fransua M, Flood M (2019). Lower gastrointestinal Kaposi sarcoma in HIV/AIDS: a diagnostic challenge. Gastrointest Tumors.

[11] Schneider JW, Dittmer DP (2017). Diagnosis and treatment of Kaposi sarcoma. Am J Clin Dermatol.

[12] Amerson E, Woodruff CM, Forrestel A (2016). Accuracy of clinical suspicion and pathologic diagnosis of Kaposi sarcoma in East Africa. J Acquir Immune Defic Syndr.

[13] Arruda É, Jacome AA, Toscano AL (2014). Consensus of the Brazilian Society of Infectious Diseases and Brazilian Society of Clinical Oncology on the management and treatment of Kaposi's sarcoma. Braz J Infect Dis.

[14] Studemeister A (2011). Cytomegalovirus proctitis: a rare and disregarded sexually transmitted disease. Sex Transm Dis.

[15] Workowski KA, Bachmann LH, Chan PA (2021). Sexually transmitted infections treatment guidelines, 2021. MMWR Recomm Rep.

